# How to Fairly Allocate Scarce Medical Resources: Ethical Argumentation under Scrutiny by Health Professionals and Lay People

**DOI:** 10.1371/journal.pone.0159086

**Published:** 2016-07-27

**Authors:** Pius Krütli, Thomas Rosemann, Kjell Y. Törnblom, Timo Smieszek

**Affiliations:** 1 Transdisciplinarity Lab (TdLab), Department of Environmental Systems Science, ETH Zurich, Switzerland; 2 Institute of Primary Care, University of Zurich, Switzerland; 3 Modelling and Economics Unit, Statistics, Modelling, and Economics Department, Public Health England, London, United Kingdom; 4 MRC Centre for Outbreak Analysis and Modelling, Department of Infectious Disease Epidemiology, Imperial College School of Public Health, London, United Kingdom; 5 Center for Infectious Disease Dynamics, The Pennsylvania State University, University Park, PA, United States of America; Mahidol-Oxford Tropical Medicine Research Unit, THAILAND

## Abstract

**Background:**

Societies are facing medical resource scarcities, inter alia due to increased life expectancy and limited health budgets and also due to temporal or continuous physical shortages of resources like donor organs. This makes it challenging to meet the medical needs of all. Ethicists provide normative guidance for how to fairly allocate scarce medical resources, but legitimate decisions require additionally information regarding what the general public considers to be fair. The purpose of this study was to explore how lay people, general practitioners, medical students and other health professionals evaluate the fairness of ten allocation principles for scarce medical resources: ‘sickest first’, ‘waiting list’, ‘prognosis’, ‘behaviour’ (i.e., those who engage in risky behaviour should not be prioritized), ‘instrumental value’ (e.g., health care workers should be favoured during epidemics), ‘combination of criteria’ (i.e., a sequence of the ‘youngest first’, ‘prognosis’, and ‘lottery’ principles), ‘reciprocity’ (i.e., those who provided services to the society in the past should be rewarded), ‘youngest first’, ‘lottery’, and ‘monetary contribution’.

**Methods:**

1,267 respondents to an online questionnaire were confronted with hypothetical situations of scarcity regarding (i) donor organs, (ii) hospital beds during an epidemic, and (iii) joint replacements. Nine allocation principles were evaluated in terms of fairness for each type of scarcity along 7-point Likert scales. The relationship between demographic factors (gender, age, religiosity, political orientation, and health status) and fairness evaluations was modelled with logistic regression.

**Results:**

Medical background was a major predictor of fairness evaluations. While general practitioners showed different response patterns for all three allocation situations, the responses by lay people were very similar. Lay people rated ‘sickest first’ and ‘waiting list’ on top of all allocation principles—e.g., for donor organs 83.8% (95% CI: [81.2%–86.2%]) rated ‘sickest first’ as fair (‘fair’ is represented by scale points 5–7), and 69.5% [66.2%–72.4%] rated ‘waiting list’ as fair. The corresponding results for general practitioners: ‘prognosis’ 79.7% [74.2%–84.9%], ‘combination of criteria’ 72.6% [66.4%–78.5%], and ‘sickest first’ 74.5% [68.6%–80.1%); these were the highest-rated allocation principles for donor organs allocation. Interestingly, only 44.3% [37.7%–50.9%] of the general practitioners rated ‘instrumental value’ as fair for the allocation of hospital beds during a flu epidemic. The fairness evaluations by general practitioners obtained for joint replacements: ‘sickest first’ 84.0% [78.8%–88.6%], ‘combination of criteria’ 65.6% [59.2%–71.8%], and ‘prognosis’ 63.7% [57.1%–70.0%]. ‘Lottery’, ‘reciprocity’, ‘instrumental value’, and ‘monetary contribution’ were considered very unfair allocation principles by both groups. Medical students’ ratings were similar to those of general practitioners, and the ratings by other health professionals resembled those of lay people.

**Conclusions:**

Results are partly at odds with current conclusions proposed by some ethicists. A number of ethicists reject ‘sickest first’ and ‘waiting list’ as morally unjustifiable allocation principles, whereas those allocation principles received the highest fairness endorsements by lay people and to some extent also by health professionals. Decision makers are advised to consider whether or not to give ethicists, health professionals, and the general public an equal voice when attempting to arrive at maximally endorsed allocations of scarce medical resources.

## Introduction

The Universal Declaration of Human Rights [1] and its specifications in the International Covenant on Economic, Social, and Cultural Rights, Art. 12, adjudges everyone „the right […] to the enjoyment of the highest attainable standard of physical and mental health” [2]. This provision includes access to all the medical resources needed to live up to that standard [3]. However, societies are facing situations when medical resources are scarce, and access to means of prevention, diagnosis, and treatment of those in need is not always guaranteed. Insufficient supply of medical resources is obvious in many developing countries where basic services are widely lacking [4–5]. Yet, also well-off countries are confronted by scarcities of medical resources such as donor organs, hospital beds during epidemics or after severe disasters, or unusually expensive drugs like Sofosbuvir to cure HCV infection [6] or Myozyme to manage Pompe disease [7].

Notwithstanding the need to reduce scarcities of critical medical resources worldwide, existing shortages necessitate principles and rules prescribing how to allocate available medical services among the needy. Explicit rules addressing whom to prioritize are in place in many countries regarding certain life-saving resources like donor organs and drugs against pandemic influenza (Box 1).

**Box 1. Allocation of scarce medical resources in Switzerland: current regulations and involvement of ethicists in the formulation of these regulations**

**Example 1: Prioritizing individuals during an influenza pandemic**

The Regulation on the Control of Communicable Diseases of Humans (Regulation 818.101.1; enacted 1 January 2016; replaced Regulation 818.101.23) sets the rules for prioritizing individuals in situations when vaccines effective against pandemic influenza (as well as other critical drugs to treat communicable diseases) might be scarce.

Any prioritization based on this regulation has to reflect generally accepted medical and ethical criteria as well as economic and societal concerns. The following groups are explicitly mentioned as eligible for prioritisation:

1. health care workers;
2. individuals, who have a higher risk of complications and adverse outcomes of the disease than others;
3. individuals, who contribute to crucial services such as inner and national security, transport, communication as well as energy, water, and food supply.

Accordingly, individuals can be prioritized according to their *instrumental value* and according to their individual *prognosis* (cf. Table 1).

The Swiss Federal Office for Public Health (FOPH, which was in charge of formulating the current and the preceding regulation) actively reflected and incorporated ethical arguments and positions coming from the Swiss National Advisory Commission on Biomedical Ethics. Academic ethicist Urs Thurnheer (Karlsruhe University of Education) was actively involved in preparatory work which fed into the regulation on pandemic influenza preceding the current regulation (personal communication [8]).

**Example 2: Allocating donor organs**

The Regulation on the Allocation of Organs for Transplantation (Regulation 810.212.4; enacted 16 March 2007; version of 1 May 2016) governs the allocation of the following donor organs: heart, lung, liver, kidney, pancreas, small intestine. Allocation rules are based on multiple criteria and differ (slightly) for different organs.

In summary,

1. medical urgency: first priority is given to patients whose life would be at immediate risk if they would not receive the organ;
2. medical benefit: second priority is given to patients, for whom the greatest medical benefit is expected.

If two (or more) patients should have the same priority according to these criteria, the following criteria shall also be taken into consideration: medical urgency, waiting time, extended waiting time due to the patient’s blood group, fit of the tissue characteristics.

Regulation 810.212.4 also uses a combination of fairness criteria with the *sickest first* principle (cf. Table 1) being the most important criterion, *prognosis* being the second most important criterion. Other principles, including the *waiting list* (cf. Table 1), only play a role if at least two patients have the same priority according to the *sickest first* and *prognosis* principles.

Upon defining the Swiss Federal Office for Public Health commissioned an expert’s report by ethicist Beat Sitter-Liver (University of Fribourg) [9] which was used as a foundation for phrasing the Regulation on the Allocation of Organs for Transplantation (personal communication [10]). Among more than 70 stakeholders, three institutions representing academic ethics (the Center for Ethics, University of Zurich; the Institute for Applied Ethics and Medical Ethics, University of Basel; the Swiss National Advisory Commission on Biomedical Ethics) were actively involved in the consultation process, which resulted in the enactment of the regulation.

Ethicists offer moral guidance for how to fairly allocate scarce medical resources, e.g., [11–13], and a number of allocation principles have been defined and balanced against each other. Persad et al. [14], for example, discuss eight principles: lottery, waiting list, sickest first, youngest first, number of lives saved, prognosis, instrumental value, and reciprocity (Table 1). In their view, most principles are fair, except for waiting lists and the sickest first principle, both of which were rejected as morally unjustifiable. The authors argue that waiting lists favour well-off people and are susceptible to corruption, while the sickest first principle ignores a patient’s prognosis and favours today’s sickest individuals over those who might be even worse-off in the future. However, medical allocation has been widely and controversially discussed in bioethics and philosophy, e.g., [15–25], and Persad et al.’s positions are disputed in many ways, e.g., [26], yet it is out of the scope of this paper to substantially contribute to this ethical discussion. In this paper, we build mainly on the substantial set of ethical principles described by Persad and colleagues.

**Table 1:** Allocation principles.

Allocation principle	Abbreviation	Description	Pros	Cons	Complete lives system
Sickest first	SICK	Prioritizes the sickest, i.e., those who have greatest need for treatment at a specific moment in time.	Intuitively obvious; sickest are also worst-off	Ignores post-treatment prognosis	Excluded
Waiting list	ORDR	Allocates services according to the individual’s position on the waiting list. Also known as ‘first-come, first-served’ principle.	Equality of opportunities; no discontinued interventions	Ignores relevant differences between individuals; favours the well-off; susceptible to corruption	Excluded
Prognosis	SURV	Prioritizes those with favourable prognosis, hence, those with the highest survival probability and duration.	Intuitively obvious; saves most life years	Does not consider distribution and number of lives saved	Included
Behaviour	BHAV	Prioritizes those who did not engage in risky behaviours that caused their condition or affected it negatively.	Promotes healthy life style; promotes individual responsibility	Reasons for individual behaviour ignored; conflict with liberty rights	Not considered
Instrumental value	IMPF	Prioritizes those who’s function is essential to keep up fundamental services, e.g., health care professionals. Relevant, e.g., during pandemics.	Serves saving most lives	Can encourage abuse of system	Included under certain conditions
Combination of criteria	COMB	This allocation scheme includes a combination of criteria such as age (youngest first), prognosis and lottery.	Considers several morally relevant criteria; appropriate distributive justice	Discriminates older people	Subset of Complete lives system approach
Youngest first	YONG	Prioritizes young over old individuals.	Prioritizes worst-off; hard to corrupt	Ignores relevant other principles	Included
Lottery	RAND	Allocates medical services randomly among those who are in need of treatment.	Equal opportunities; little knowledge about recipients needed; easy to handle; resistant against corruption	Blind against other factors; treating people equally often fails to treat them as equals	Included
Reciprocity	SERV	Prioritizes those who have voluntarily provided societal services in the past.	Justice to people who have provided contributions in the past	Requires complex inquiries	Included under certain conditions
Monetary contribution	MONY	Prioritizes those who contribute to the costs of medical treatment.	Relieves public health system; reduces costs; reflects common societal principle that those who need more pay more	Favours wealthy people; undermines societal solidarity; makes allocation to worst-offs impossible	Not considered

Source: adapted from Persad et al. (2009, p. 424) [14]. Column ‘Abbreviation’ corresponds to Fig 1 and Table 2 in Results section.

In contrast to the prescriptive approach in ethics, the social psychological focus is descriptive and explores people’s subjective perceptions of justice–“justice is in the eye of the beholder” [27]. Thus, opinions about what is a fair (in this article, the terms ‘fair’ and ‘just’ are used interchangeably) allocation of social resources vary with the context and may differ between individuals, groups and cultures [28]. The most commonly discussed principles are allocation according to needs, contributions (of effort, ability, and/or results), or equal amounts to all. See [29] for an overview of additional ‘generic’ allocation principles (into which most of the medical resource allocation principles focused by Persad et al. and other researches [14, 30], for example, can be translated).

Fairness judgments of resource allocation principles may be affected by a large number of factors [31] such as the allocated resource, per se [32], the social relationship [33], and the societal context [34–35]. Even though studies within the empirical medical research tradition typically focused on justice conceptions separately of patients, clinicians, lay people, and medical students, comparisons among these categories of people within the framework of one single study are lacking (like in the cases of [36, 30]). Previous studies have shown that different group identities appeared to affect moral judgments and behaviour differently, e.g., [37–38]. Existing empirical research has also focused on the impact of individual recipient characteristics like gender, age, life-style, health status, etc. [39–46], as well as on specific priority principles and allocation situations [47–50].

Finally, and of special importance in the context of the study reported here, there is also a lack of comprehensive empirical studies that combine and compare the descriptive and prescriptive approaches [51]. While ethics provide the moral fundament, enforceability of rules in democratic societies require majority endorsement as well as consensus among stakeholder groups. Thus, the descriptive and prescriptive approaches may complement each other to better guide decisions about social resource allocation.

The major objective of the study reported here was to study how (a) four categories of people (lay people, general practitioners, medical students and other health professionals) evaluate the fairness of (b) ten allocation principles (see methods section) for (c) three scarce medical resources (donor organs, hospital beds, joint replacements). We (d) compared our empirically obtained fairness evaluations of the ten allocation principles with those derived ‘prescriptively/ethically’ by Persad et al. [14] to reveal in/consistencies between the their version of the ethical (philosophical) approach and the empirical (descriptive/social psychological) approach. Finally, we (e) explored whether certain individual characteristics might account for observed variations in fairness perceptions.

## Methods

An online survey containing 99 questions was conducted between December 2, 2013 and May 31, 2014. To avoid bias by row effects, questions were presented in random order, and the three types of scarce resources were randomized as well. A total of 1,790 respondents accessed the questionnaire, and 1,267 (71%) answered all questions. Incomplete datasets were excluded from the analysis. The questionnaire was pre-tested and discussed with peers for clarity of questions and logical, bias-reducing order among questions. For the current study only a subset of questionnaire items were used. The respective dataset can be found as S1 Dataset and S1 Text (see also S1 File).

### Respondents

Participants were recruited from three predefined pools: a market research panel (MRP), general practitioners (GP), and medical students (MS). The MRP consisted of a sample of the 25–65 year old population from the German-speaking part of Switzerland (representative in terms of age- and gender-distribution). Most GPs were recruited from the cantons of Zurich and Lucerne (1) via invitation letters to all 1,415 GPs in the canton of Zurich who were enrolled with the Swiss Medical Association, (2) by asking medical networks to send invitation emails to their associated GPs (a pool of approx. 250 GPs), and (3) advertising the study in Swiss medical newsletters. MSs were approached by sending invitation emails to all students enrolled at the Faculty of Medicine at the University of Zurich. Incentives were offered to increase response rates (lotteries with 10 prices valued CHF 300 each for GPs and MSs and EUR 3 payments for all online panellists (paid by the MRP provider)).

**Fig 1:**
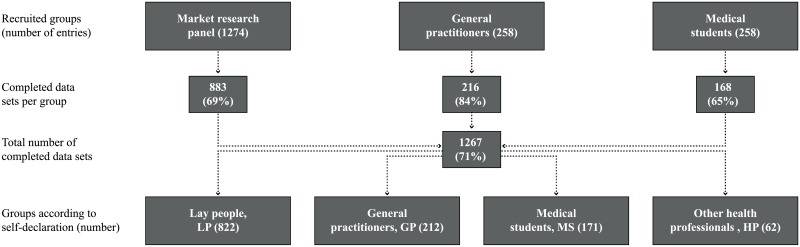
Study sample. Top row: three different sources of respondents; bottom row: self-declared medical background.

Respondents declared themselves as physicians (GP), medical students (MS), other health professionals (HP), or others (i.e., lay people, LP), see Fig 1. Socio-demographic profiles of these four groups are provided as S1 Table.

### Questionnaire

Participants were provided descriptions of three hypothetical situations in which the three types of scarce medical resources were to be allocated, i.e. (i) *donor organs*–an inelastic resource, (ii) *hospital beds during a flu epidemic*–an elastic resource, and (iii) *joint replacements*–an elastic resource (Table 2). The respondents were asked to give their advice on how they thought the three resources *should* be allocated (thus, generating descriptions of respondents’ prescriptive views) choosing from a list of nine allocation principles (i.e., selection of the most fair principle), each of which they also rated in terms of fairness along 7-point Likert scales ranging from 1 (totally unjust) to 7 (totally just) (Table 2). Not all allocation principles were included in every situation. Additional data included participants’ gender, age, religiosity, political orientation, and health status. Screen shots of the online survey are provided as S1 File.

**Table 2:** Three hypothetical situations of scarce medical resource allocation, and their respective nine allocation principle alternatives.

**Situation A: Donor organs**	**Situation B: Hospital beds during epidemic**	**Situation C: Joint replacements**
One hundred organs (kidneys) are available yearly from voluntary and eligible donors. A team of consultants is responsible for the allocation of the 100 donated kidneys to some of the 500 individuals who are in need of a kidney transplant. For convenience, we assume that the kidneys are equally tolerable to all 500 individuals.	A very severe flu epidemic hits a mid-sized town (approx. 50,000 inhabitants) in Switzerland and, as a consequence, 2,500 individuals need hospital care. There are, however, only 500 hospital beds available. A team of consultants will allocate the 500 hospital beds to some of the 2,500 individuals in need.	In Switzerland, there are 5,000 individuals who are waiting for a life-quality enhancing treatment, e.g., hip-joint replacement. Thus, they don’t suffer from a life-threatening condition. This treatment is very expensive, and only 1,000 hip-joint replacements can be provided. A team of consultants will allocate the 1,000 replacements to some of the 5,000 individuals who are in need of it.
**Problem (Question): In your opinion, how should the team of consultants proceed?**		
**The 100 kidneys should be allocated:**	**The 500 beds should be allocated:**	**The 1,000 hip-joints should be allocated:**
to the sickest^a^ individuals, i.e., those, who need the organ most urgently [SICK]	to the sickest individuals [SICK]	to the sickest individuals (i.e., those whose hip problem results in the most severe reduction in life-quality) [SICK]
according to the order of registration for a donor organ (i.e., those with the longest wait are prioritized) [ORDR]	according to the order of falling sick (i.e., those who have been ill the longest are prioritized) [ORDR]	according to the order of registration for surgery (i.e., those with the longest wait are prioritized) [ORDR]
by prioritizing those who are likely to survive the longest because of the organ transplant [SURV]	by prioritizing those who are most likely to survive the infection as a result of hospital care [SURV]	by prioritizing those with the longest life expectancy [SURV]
by favouring those, who have not become by own fault a medical emergency [BHAV]	by prioritizing those, who have essential roles for keeping society operational (e.g., hospital staff) [IMPF]	by prioritizing those, whose life-quality improvement needs are not self-inflicted [BHAV]
according to a combination of criteria: age (youngest first), prognosis (longest survival with organ transplant), and by chance (drawing lots) [COMB]	according to a combination of criteria: age (youngest first), prognosis (longest survival), and by chance (drawing lots) [COMB]	according to a combination of criteria: age (youngest first), prognosis (longest life expectancy based on general state of health), and by chance (drawing lots) [COMB]
according to age, prioritizing young individuals [YONG]	according to age, prioritizing young individuals [YONG]	according to age, prioritizing young individuals. [YONG]
randomly, e.g., via a lottery [RAND]	randomly, e.g., via a lottery [RAND]	randomly, e.g., via a lottery [RAND]
by prioritizing those who contributed in the past to the common good (e.g., by volunteering) [SERV]	by prioritizing those who contributed in the past to the common good (e.g., by volunteering) [SERV]	by prioritizing those who contributed in the past to the common good (e.g., by volunteering) [SERV]
preferably to those who contribute substantially to the costs of the treatment [MONY]	preferably to those who contribute substantially to the costs of the treatment [MONY]	preferably to those who contribute substantially to the costs of the treatment [MONY]

Acronyms in brackets correspond to those in Table 1. Translations from German by the authors, see also S1 File.

^a^ Persad et al. define ‘sickest first’ as”*prioritizing the person who will die soonest if she does not receive an organ*”. Our definition is slightly different: ”*those who need the organ most urgently*” should receive the organ. As ‘urgency’ may be defined in different ways, apart from helping those ‘who will die soonest’, our data may not be unambiguously compared.

### Ethics Statement

The research protocol was submitted to the ETH Ethics Commission for review and approval. The executive secretary reviewed the protocol and decided that, according to the law and pertinent regulations, a full evaluation was not required (decision 19 Sept 2013, EK 2013-N-44), because pure survey research of this kind presents no risk of harm to participants, all participants were at least 18 years old, and also because there were no data protection concerns as no personally identifiable information (PII) were about to be collected.

Following our research protocol, all potential participants were informed about the purpose of this research and the expected duration of participation. They were informed about whom to contact for questions and concerns regarding the study. We explained that all information was collected in a fully anonymous manner. Finally, participation was voluntary and participants had the opportunity to stop participation at all times before submitting the fully completed online questionnaire. All survey questions relevant to this paper can be found as S1 File.

Participation implied consent and—in line with our research protocol submitted to the ETH Ethics Commission—we did not document informed consent because the research did not involve more than minimal risk and a signed consent document would have been the only record linking participant and data (PII), and, thus, making a fully anonymous research design impossible.

### Statistical Analyses

**Fig 2:**
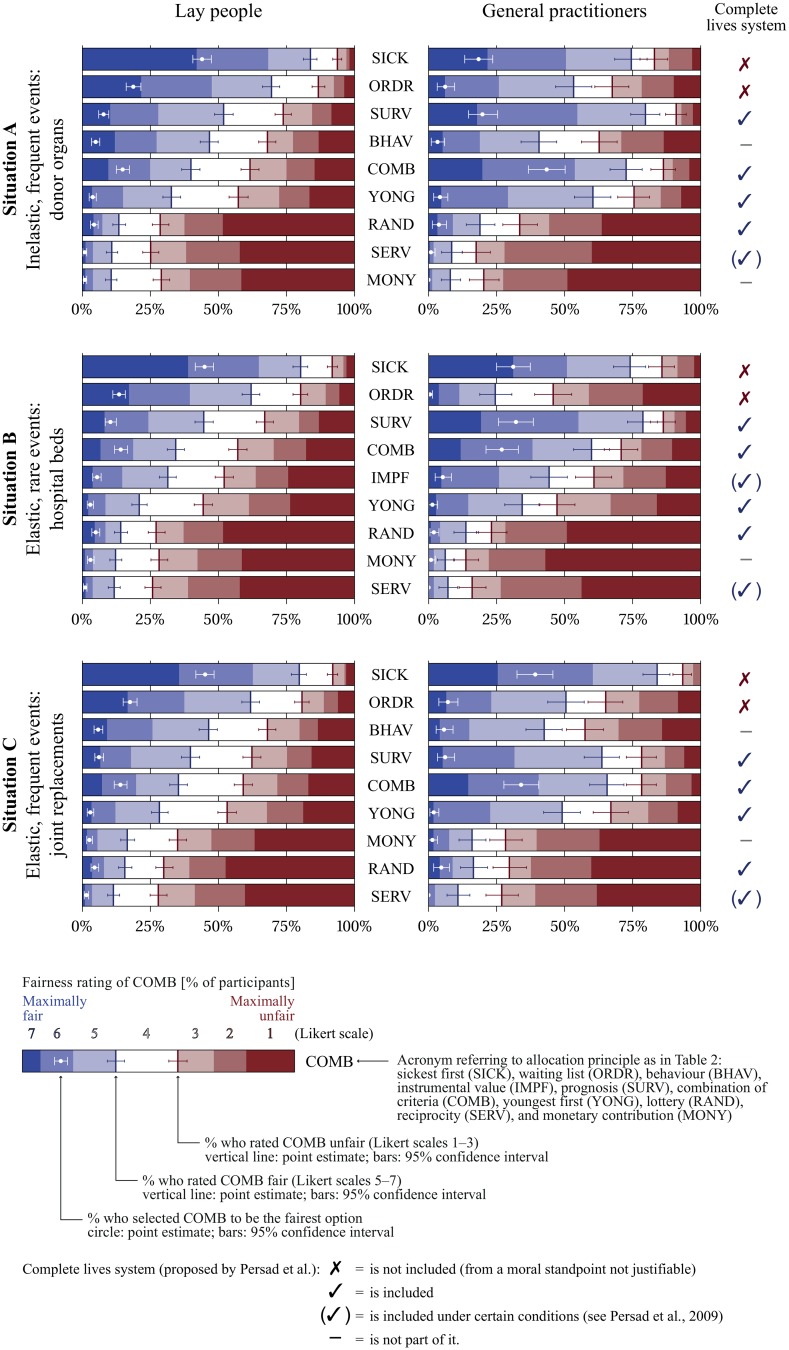
Fairness ratings and forced choice of one single principle (percentage and 95% CI) by lay people versus general practitioners for three situations of scarce medical resource allocation. For the groups medical students and other health professionals see S1 Fig).

Fig 2 provides the following information for the allocation principles for each of the three situations: (a) the frequency distribution of the Likert categories (totally just to totally unjust), (b) the proportion of participants favouring the respective principle over all other principles, (c) bootstrap 95% confidence intervals for that proportion as well as for the estimates of the borders between categories ‘just’ (Likert scales 5–7) and ‘unjust’ (1–3). The figure was rendered using ggplot2 0.9.3.1, and the confidence intervals were simulated using Python code executed with Enthought Canopy 1.1.0. Group-wise means and standard deviations are provided as S2 Table.

Logistic regression models were estimated for all allocation principles as dependent variables. The answer scales were merged into two remaining categories: (1) ‘just’ (Likert scales 5–7); (0) ‘other’ (1–4). For reasons of comparability, we estimated models with identical structure for all questions, including medical background, gender, age, religiosity, political orientation, and health state as independent variables (previous research by Skitka and Tetlock [39] suggested that these demographic variables may affect justice judgements). Regression models were estimated using IBM SPSS Version 22.

## Results

### Descriptive Statistics

Substantial differences between GPs and LPs were obtained for all three allocation situations in terms of both fairness ratings and the fairest of all allocation rules (Fig 2). While GPs show different response patterns for all the three allocation situations, the responses by LP are very similar in all three situations. The general response pattern of MSs is similar to that of GPs, while the responses by HPs resemble those by LPs (see S1 Fig).

LPs rated the *sickest first* principle and *waiting list* highest in all three situations. *Lottery*, *monetary contribution*, and *reciprocity* received the lowest ratings and are, hence, considered the most unjust allocation principles. All other principles were rated neither fair nor unfair—except in situation B. When LPs had to name the fairest principle, *sickest first* was chosen unequivocally for all three situations.

GP’s preferences differed for all three situations. *Prognosis*, *sickest first*, and *combination of criteria* were the highest-rated allocation principles for situation A. In contrast to LPs, *waiting list* was contested, while *youngest first* obtained solid support (majority of positive ratings). *Behaviour* was a contested principle in situation A, and all other principles were considered unfair allocation principles in all situations. *Sickest first*, *prognosis*, and *combination of criteria* obtained clear majorities in situation B, whereas *waiting list*, *youngest first* and, interestingly, *instrumental value* were contested fairness principles during a flu epidemic. In situation C, we observe a high rating for the *sickest first* principle. In addition, *prognosis* and *combination of criteria* were considered fair principles, while *waiting list*, *behaviour* and *youngest first* were contested. When GPs had to choose the fairest of all principles, they again answered differently for all three situations. They clearly favoured *combination of criteria in* situation A. In situation B, *sickest first*, *prognosis*, and *combination of criteria* were chosen by about the same proportion of participants. In situation C, *sickest first* and *combination of criteria* were the most favoured principles.

### Logistic Regression Model

Table 3 shows parameter estimates from the logistic regression models (reported are odds ratios, OR, and the corresponding 95% confidence intervals, CI). All subsequently reported group differences were statistically significant with p <0.05.

**Table 3:** Odds ratios (95% CIs) of allocation principles by explanatory variables such as medical background, gender, age, religion, political orientation, and health state in binary logistic regression model.

Allocation principle	Odds ratio, OR (95% Confidence interval, CI)								
	Medical background^a^			Gender	Age	Religion^b^		Pol. Orientation	Subjective Health State
	Medical Students	General Practitioners	Other health professionals	Male	Years	Not religious	Unknown	Left-right	Very bad-very good
**Situation A:** Inelastic/frequent events (donor organs)									
SICK	1.07 (0.62–1.83)	**0.47 (0.31–0.72)**	**0.52 (0.28–0.97)**	**0.70 (0.51–0.95)**	1.01 (1.00–1.03)	0.75 (0.55–1.02)	0.74 (0.42–1.30)	0.97 (0.91–1.04)	1.03 (0.90–1.17)
ORDR	0.79 (0.51–1.22)	**0.44 (0.31–0.63)**	0.76 (0.44–1.33)	0.86 (0.67–1.10)	1.00 (0.99–1.02)	0.89 (0.69–1.15)	**0.55 (0.35–0.87)**	0.99 (0.94–1.05)	1.10 (0.99–1.22)
SURV	**5.77 (3.45–9.66)**	**3.35 (2.26–4.98)**	**2.81 (1.55–5.08)**	1.14 (0.89–1.46)	1.00 (0.99–1.01)	0.90 (0.70–1.16)	1.04 (0.65–1.69)	1.03 (0.97–1.09)	1.04 (0.94–1.15)
**BHAV**	**1.87 (1.23–2.86)**	0.75 (0.53–1.06)	1.57 (0.92–2.67)	0.83 (0.66–1.05)	1.01 (1.00–1.02)	1.00 (0.79–1.27)	0.82 (0.52–1.29)	**1.13 (1.08–1.20)**	1.07 (0.97–1.18)
COMB	**3.71 (2.34–5.87)**	**3.79 (2.62–5.49)**	**2.13 (1.25–3.64)**	1.11 (0.87–1.42)	0.99 (0.98–1.00)	0.95 (0.74–1.22)	1.11 (0.69–1.77)	**0.94 (0.89–1.00)**	**1.12 (1.01–1.24)**
YONG	**2.13 (1.39–3.27)**	**2.90 (2.04–4.13)**	**1.90 (1.12–3.22)**	1.12 (0.88–1.43)	1.00 (0.98–1.01)	**0.78 (0.61–1.00)**	0.80 (0.50–1.27)	1.03 (0.98–1.09)	1.10 (1.00–1.22)
RAND	1.33 (0.77–2.30)	1.47 (0.92–2.34)	0.95 (0.41–2.16)	**1.50 (1.08–2.08)**	0.99 (0.97–1.01)	1.29 (0.93–1.79)	0.74 (0.36–1.50)	**0.88 (0.82–0.95)**	1.03 (0.90–1.19)
SERV	1.36 (0.69–2.67)	0.75 (0.42–1.35)	0.99 (0.41–2.41)	1.37 (0.94–2.01)	1.00 (0.99–1.02)	0.85 (0.57–1.26)	1.23 (0.63–2.40)	1.05 (0.96–1.14)	0.90 (0.78–1.05)
MONY	0.70 (0.31–1.55)	0.69 (0.38–1.26)	0.89 (0.34–2.33)	1.40 (0.93–2.11)	1.00 (0.98–1.02)	0.83 (0.55–1.27)	1.69 (0.87–3.26)	**1.21 (1.10–1.33)**	1.12 (0.94–1.33)
**Situation B:** Elastic/rare events (hospital beds)									
SICK	**1.99 (1.12–3.50)**	**0.57 (0.38–0.86)**	0.57 (0.32–1.04)	**0.73 (0.54–0.98)**	**1.02 (1.00–1.03)**	1.03 (0.77–1.39)	0.99 (0.57–1.72)	0.94 (0.89–1.01)	1.08 (0.96–1.22)
ORDR	**0.49 (0.32–0.75)**	**0.19 (0.13–0.27)**	0.96 (0.56–1.66)	**0.78 (0.61–0.99)**	1.00 (0.99–1.02)	0.87 (0.68–1.11)	0.95 (0.60–1.50)	**1.06 (1.01–1.12)**	1.09 (0.98–1.20)
SURV	**4.03 (2.52–6.44)**	**4.68 (3.16–6.93)**	1.43 (0.84–2.42)	1.20 (0.94–1.53)	0.99 (0.98–1.00)	1.00 (0.78–1.28)	0.73 (0.46–1.17)	1.00 (0.95–1.06)	1.03 (0.93–1.14)
**IMPF**	**2.35 (1.51–3.66)**	1.38 (0.97–1.95)	1.50 (0.87–2.59)	1.24 (0.97–1.59)	**1.02 (1.00–1.03)**	**0.67 (0.52–0.86)**	0.87 (0.55–1.39)	1.04 (0.99–1.10)	1.00 (0.90–1.11)
COMB	**1.93 (1.26–2.97)**	**2.60 (1.83–3.70)**	**2.24 (1.31–3.81)**	**1.34 (1.05–1.71)**	**0.99 (0.98–1.00)**	0.82 (0.64–1.05)	0.94 (0.59–1.49)	**0.93 (0.88–0.99)**	**1.16 (1.04–1.29)**
YONG	1.40 (0.86–2.27)	**1.71 (1.17–2.49)**	1.44 (0.79–2.64)	1.30 (0.99–1.72)	1.00 (0.99–1.01)	**0.75 (0.56–0.99)**	0.86 (0.51–1.45)	1.01 (0.95–1.08)	1.07 (0.95–1.21)
RAND	1.05 (0.62–1.80)	0.98 (0.59–1.60)	1.20 (0.59–2.47)	1.14 (0.82–1.57)	0.98 (0.97–1.00)	1.12 (0.81–1.56)	0.82 (0.42–1.58)	**0.87 (0.80–0.94)**	1.09 (0.94–1.26)
SERV	1.28 (0.65–2.49)	**0.48 (0.26–0.89)**	1.35 (0.61–2.97)	**1.65 (1.13–2.42)**	1.01 (0.99–1.02)	0.75 (0.51–1.10)	1.13 (0.58–2.19)	1.02 (0.94–1.11)	0.99 (0.85–1.15)
MONY	**0.32 (0.13–0.79)**	0.50 (0.26–0.96)	0.67 (0.26–1.77)	1.03 (0.69–1.52)	1.00 (0.98–1.02)	1.12 (0.74–1.69)	**2.41 (1.29–4.52)**	**1.27 (1.16–1.39)**	**1.24 (1.04–1.47)**
**Situation C:** Elastic/frequent events (joint replacement)									
SICK	**4.06 (2.08–7.93)**	1.06 (0.67–1.68)	0.97 (0.49–1.94)	**0.56 (0.41–0.76)**	**1.02 (1.01–1.04)**	1.06 (0.78–1.46)	0.83 (0.47–1.45)	1.00 (0.93–1.07)	**1.15 (1.01–1.30)**
ORDR	0.86 (0.56–1.32)	**0.61 (0.44–0.87)**	0.86 (0.50–1.47)	**0.68 (0.54–0.86)**	1.00 (0.99–1.01)	0.91 (0.71–1.16)	0.67 (0.43 1.06)	1.01 (0.95–1.06)	**1.15 (1.04–1.27)**
SURV	**2.33 (1.52–3.57)**	**2.52 (1.77–3.58)**	1.29 (0.76–2.18)	1.10 (0.87–1.39)	1.00 (0.99–1.01)	0.88 (0.69–1.12)	0.94 (0.60–1.47)	1.00 (0.94–1.05)	1.06 (0.96–1.17)
**BHAV**	**1.95 (1.28–2.99)**	0.69 (0.49–0.98)	1.38 (0.82–2.35)	0.99 (0.78–1.25)	**1.02 (1.01–1.03)**	1.06 (0.84–1.35)	1.19 (0.76–1.87)	**1.12 (1.07–1.19)**	**1.12 (1.01–1.24)**
COMB	**3.25 (2.08–5.08)**	**3.62 (2.52–5.18)**	**2.89 (1.68–4.95)**	1.19 (0.93–1.52)	**0.98 (0.97–1.00)**	0.86 (0.67–1.10)	1.10 (0.69–1.76)	0.99 (0.93–1.04)	**1.12 (1.00–1.24)**
YONG	**2.42 (1.57–3.75)**	**2.24 (1.57–3.19)**	**2.04 (1.19–3.49)**	**1.30 (1.01–1.66)**	1.00 (0.98–1.01)	**0.75 (0.58–0.96)**	**0.54 (0.33–0.90)**	1.02 (0.97–1.08)	1.05 (0.95–1.17)
RAND	1.23 (0.74–2.02)	1.23 (0.77–1.96)	1.34 (0.68–2.62)	1.14 (0.84–1.55)	**0.98 (0.96–0.99)**	1.14 (0.84–1.56)	1.12 (0.63–1.98)	**0.91 (0.84–0.97)**	1.06 (0.93–1.21)
SERV	0.79 (0.39–1.61)	0.82 (0.48–1.41)	0.85 (0.35–2.05)	1.08 (0.74–1.57)	1.00 (0.98–1.02)	**0.58 (0.39–0.86)**	0.83 (0.41–1.68)	1.06 (0.98–1.16)	1.07 (0.91–1.26)
MONY	1.06 (0.60–1.85)	1.02 (0.64–1.64)	0.88 (0.40–1.93)	**1.41 (1.02–1.94)**	0.99 (0.97–1.00)	0.83 (0.60–1.16)	1.52 (0.88–2.61)	**1.22 (1.13–1.31)**	1.13 (0.98–1.30)

^a^ reference category is lay people

^b^ reference category is religious

bold: sig. p <0.05.

Medical background was the most influential independent variable. Major differences can be observed between reference groups LP and GP, LP and MS, and, partially, between the LP and HP groups.

GPs were between 1.71 and 4.68 times (OR) more likely than LPs to choose *prognosis*, *combination of criteria*, and *youngest first*. Further, GPs were less likely than LPs to choose *waiting list* and *sickest first* except in situation C, and *reciprocity* in situation B (OR 0.19–0.61). Similarly, like GPs, MSs (and HPs, but to a lesser degree) deviated from LP’s choices. However, MSs were almost six times as likely as LPs to choose *prognosis* in situation A. Further, they were twice as likely (situation B) and four times as likely (situation C) than LPs to choose *sickest first*. Most differences between HPs and LPs occurred in the context of situation A.

Men showed greater preferences than women for *lottery* in situation A (OR = 1.50), *combination of criteria* (OR = 1.34) and *instrumental value* (OR = 1.65) in situation B, and *youngest first* (OR = 1.30) as well as *monetary contribution* (OR = 1.41) in situation C, while women considered *sickest first* principle to be more fair than men in all situations. They thought *waiting list* in situations B and C to be a more fair allocation principle than did men.

No significant age effect was observed in situation A, while in situations B and C increasing age correlated with an increased preference for *sickest first* (OR = 1.02), as well as for *instrumental value* (OR = 1.02) in situation B, and *behaviour* (OR = 1.02) in situation C. The opposite was observed for *combination of criteria* in situations B (OR = 0.99) and C (OR = 0.98), and *lottery* (OR = 0.98) in situation and C.

Participants who declared themselves religious showed clearer preferences than non-religious participants for *youngest first* in all three situations, for *instrumental value* in situation B (OR = 0.67), and for *reciprocity* in situation C (OR = 0.58). Those who were uncertain regarding their religiosity also deviated from the religious participants (ORDR, OR(A) = 0.55; MONY, OR(B) = 2.41; YONG, OR(C) = 0.54).

Political orientation had an effect on *monetary contribution* in all three situations and on *behaviour* in situations A and C: the more a participant was leaning towards the political right, the more likely s/he was to consider these principles to be fair. The opposite effect regarding the left-right spectrum (but to a lesser extent) was observed for *combination of criteria* in situations A and B and for *lottery* in all situations: the more a participant was left-oriented, the more likely s/he was to consider these principles to be fair.

Finally, participants’ self-declared health state impacted their evaluation pattern in situation C: *sickest first*, *waiting list*, *behaviour* and *combination of criteria* were significantly more preferred by healthier participants. The same effect was observed for *combination of criteria* in situations A and B, and for *monetary contribution* in situation B (OR = 1.24).

## Discussion

Our data suggest that fairness ratings covary with (i) the rater’s medical background, (ii) the allocated resource, and with (iii) the individual factors gender, age, religiosity, political orientation, and health status. The *sickest first* principle was clearly prioritized by LPs (see also [48]) in all three allocation situations and more so by females than by males. In theory, the *sickest first* principle favours the worst-off (i.e., those whose life may be at stake) and is equivalent to the *need* principle which is considered most fair when the recipient’s welfare is prioritized [33]. *Sickest first*, albeit to a slightly lesser extent, was also highly endorsed by HPs, MSs and GPs. Yet, it competed (especially in the donor organ situation) with *prognosis* and with *combination of criteria*.

*Sickest first* was age-dependent in situations B (hospital beds) and C (joint replacement), i.e., considered increasingly fair with advancing age of the rater—most likely because of older people’s increased mortality risk from influenza and their higher prevalence of suffering from worn-out (hip) joints.

Our empirical data do not support the normative claims by ethicists Persad et al. [14] that the *sickest first* principle is not morally justifiable. As our respondents were asked how they thought the three resources *should* be allocated (i.e., how fair the respective allocation principle is), we may assume that we have tapped their moral standpoint. If so, this may pose a challenge for ethicists (of Persad et al.’s orientation) as well as for health care administrators. However, ethicists may argue that normative requirements cannot be deduced from empirical data [52]. Whether or not this is true, it may be unwise to ignore the discrepancy between empirically tapped normative standpoints and ethicists’ moral conclusions derived on the basis of non-empirical deductions. Furthermore, moral standards may shift over time, and decisions in a democracy will not be sustainable in the long run unless legitimized by a majority.

The *waiting list* principle is also in contrast to what ethicists suggest. It is considered very fair by LP (and more so by females than by males in situations B and C) and to a lesser extent by MSs and HPs. However, this principle is contested by GPs. *Waiting list* may seem attractive at first glance, as it implies equality of opportunity [53], but the GPs may recognize its inherent shortcoming (Table 1).

*Prognosis* is top ranked in situations A and B by GPs, MSs (and HPs in situation A only). To estimate a patient’s chances of survival given a particular treatment requires the knowledge and experience possessed by GPs—and to some extent HPs and MSs as well. This may best explain the discrepancy between these three groups and LP.

Whether one should take into consideration if a person’s *behaviour* was harmful to her/his health or not is contested by respondents. Political orientation varies markedly with fairness conceptions, i.e., the more a respondent was leaning to the political right, the more likely s/he considered this principle to be fair. *Behaviour* has to do with responsibility, and it is well known that those on the right side of the political spectrum stress individual responsibility. Earlier studies also suggest that people tend to negatively sanction those who are deemed responsible for their predicament, e.g., abuse of alcohol and corresponding need for liver transplant [39–40, 44–45, 54].

*Instrumental value* is a principle that is included in several pandemic preparedness plans [55]. It prioritizes those (e.g., health care workers) who are pivotal in keeping essential services functioning. However, it is a questionable principle from a moral standpoint, because its focus is on efficiency that does not consider individual needs [17]. Likely results from the application of this principle highlight the dilemma between favouring the worst-offs (individual perspective) and saving the most lives (utilitarianism, societal perspective).

*Combination of criteria* is a principle favoured by GPs and MSs, particularly in situations A and C. Practitioners are exposed to patients on a daily basis and are well aware that ‘one-size-fits-all’ approaches may not be feasible when allocating scarce medical resources. The need for differentiation is obvious, as neither *sickest first*, *waiting list*, or *prognosis* may be adequate allocation principles for all specific situations of medical scarcity. The *combination* principle may also reflect current practice (see Box 1), particularly when donor organs are allocated. Both political orientation and health status affect the response pattern: the more left-leaning and the healthier respondents are, the more likely they are to consider a *combination of criteria* fair.

*Youngest first* is another controversial principle, as illustrated in a recent German study [49]. Only GPs in situation A consider this principle fair to a certain extent. This may reflect the controversy of giving a young person a chance to live a full life-span and at the same time not excluding old people [56–57]. Furthermore, religious (more than non-religious) respondents favour *youngest first*. Most religious people in Switzerland belong to a Abrahamic religion which implies that “there is a time for everything […] time to give birth and a time to die; a time to plant and a time to harvest what is planted” [58]. In other words, younger persons have to be granted time to “harvest”.

Counter-intuitively, neither group considers *lottery* to be fair. This principle is frequently rejected, although it is a very fair principle from a moral standpoint, as it gives everybody an equal chance/opportunity [59]. The major disadvantage, as Persad et al. [14] point out, is that *lottery* is insufficient, blind to other relevant factors. The more left-oriented respondents are, the more likely they are to consider this principle fair. This can perhaps be explained by the fact that *lottery* is a type of equality which is a major value in left-oriented groups. On the other hand, *monetary contribution* is often opted by right-oriented persons who favour individual responsibility and less government involvement. Still, in view of the fact that health care regulators may come under huge pressure to balance increasing health costs and patients’ needs (e.g., by expensive new therapies), this clear rejection of *monetary contribution* cannot be ignored.

### Limitations

The samples of participants in our study may not be representative of the populations from which they were drawn. Although we made sure that the participants from the MRP matched the age and gender distributions of the German-speaking part of Switzerland, and the fact that other demographic variables (level of education, political orientation) also seem to be balanced and in line with our expectations, we cannot rule out selection and self-selection biases.

Further, with regard to our selection of allocation principles following Persad et al. [14] (there is a slight difference in how we defined *sickest first*), we may have overlooked principles that might have been relevant and perhaps preferred by our respondents (see, e.g., [48]). Further, our participants might have responded differently given additional time to reflect more thoroughly on the presented allocation situations and on the implications of the various allocation principles

The generalizability of our findings to other populations is limited. Switzerland is among the ten wealthiest countries (GDP capita, at purchasing power parity [60]) and, hence, scarcity problems exist on a very different level and affect less people than in many other countries. As a consequence, our findings may not apply in its entirety to societies in poor parts of the world, where scarcities of basic medical resources are widespread or, for that matter, even to other wealthy countries. Diverging perceptions of what is fair are also likely to exist due to individual experiences with different healthcare systems. Further, there is ample evidence in the literature on justice, that cultural differences in fairness judgements are common [54].

Finally, comparisons between studies on principles for social resource allocation, in this case, between those with a focus on medical resources, may not be entirely valid. The reason is that respondents are not always asked to evaluate or rank order the principles in terms of the same criterion. Our study tapped prescriptive justice evaluations, while others may concern evaluations in terms of preferences, importance, endorsement, support, or stated behavioural intentions.

## Conclusions

Ethical reasoning is prescriptive and asks ‘what ought to be’. The present study complements this perspective via a social psychological, empirical description of respondents’ prescriptions. They were asked according to which principle (from a list of nine per resource) they think three resources *should* be allocated, and how just and fair they consider each one of the nine allocation principles to be. The social psychological approach involves ‘what is’, in this case what is a fair allocation of scarce medical resources according to the respondents’ subjective perceptions?

However, it may be unwise to derive normative (moral) principles from empirical results [52]. A majority opinion is no guarantee against moral wrong-doings, as the history of humankind has repeatedly shown. Nevertheless, and due to the possibility that the prescriptive preferences of the general public and the ethicists’ theoretical moral derivations may not necessarily be in agreement, a generally accepted foundation is crucial, on the basis of which allocation principles for scarce medical resources are morally justified and democratically accepted. Empirical insights cannot be ignored in the context of normative justice research, and vice versa. Ethicists as well as health care regulators need to take into consideration what people perceive as just allocation of medical resources. Lest we risk the two justice perspectives to become completely detached from each other. From an academic perspective this may not be a problem, but from a clinical and societal perspective it would clearly matter: normative claims may be considered unrealistic and even outright unfair, and may—as a result—be ignored in daily life.

We identified such a gap regarding the popular principles *sickest first* (endorsed by all groups) and *waiting list* (not favoured by GPs), both of which conflict with the ethical perspective of Persad et al. [14]. We assume that most GPs are aware of the inherent disadvantages of the *waiting list* principle (Table 1), and that the other groups would agree on less favourable fairness judgements of this principle, if they were equally well informed. On the other hand, we expect that most groups would reject Persad et al.’s [14] arguments against the *sickest first* principle as practically inapplicable, for instance and in particular their criticism of the principle’s inherent trade-off between the neediest today versus those of the future. If ethicists believe their arguments to be the most ethically beneficial, attempts to influence people’s fairness perceptions are needed in order to narrow the gap between both justice perspectives.

We think societal consensus among respondent groups is possible, even though their justice evaluations may diverge. Existing differences in opinions among different categories of stakeholders prompts us to face the problem of how to select a (or a combination of) legitimate decision maker(s) to make possible fair allocations of scarce medical resources.

In summary, our study

- widens the scope of the discussion about how to fairly allocate scarce medical resources by combining ethical reasoning and empirical data,
- is comprehensive and takes us beyond earlier empirical studies of scarce medical resource allocation, in that we include ten allocation principles, three medical resources, and four evaluation groups,
- enlightens academic as well as societal discussions, as our empirical data contradict certain ethical standpoints about major allocation principles, and
- may challenge current practice and health regulators, as it reveals major differences in fairness conceptions between different categories of people.

Considering the nature of our results we recommend that (1) a dialogue is initiated concerning the prescriptive ethicist’s and the general public’s views of justice concerning medical resource allocation, and that (2) health administrators, decision-makers, and allocators of scarce medical resources be alerted to the necessity of finding fair, efficient, and legitimate solutions. To cite Richardson “[…] ethical principles should be broadly consistent with community values but […] community values should, in turn, be subjected to ethical scrutiny, debate and revision.”, p.5 [61].

## Supporting Information

S1 Dataset Dataset used for the current study. (CSV)

S1 Fig Fairness ratings and forced choice responses (percentage and 95% CI) by medical students versus other health professionals regarding three situations of scarce medical resource allocation. (EPS)

S1 File Screenshot of the online questionnaire relevant to this paper. The file includes translated text from German.
(PDF)

S1 Table Socio-demographic profile of respondent groups. ^a^ Percentage; ^b^ 11-point scales ranging from 1 = most left to 11 = most right.
(DOC)

S2 Table Mean (M), standard deviation (SD), F-statistic, and p-values of fairness ratings of nine allocation principles by medical students, general practitioners, other health professionals, and lay people for three situations of scarce medical resource allocations. 7-point Likert scales ranging from 1 = totally unjust to 7 = totally just.
(DOC)

S1 Text Codebook of dataset used for the current study. (DOCX)
