# Supplementary material for: How to Fairly Allocate Scarce Medical Resources: Ethical Argumentation under Scrutiny by Health Professionals and Lay People
Source: PLoS One. 2016 Jul 27;11(7):e0159086. doi: 10.1371/journal.pone.0159086 (PMC4963105; doi:10.1371/journal.pone.0159086)
Supplement: S1 File — The file includes translated text from German. (PDF) [file pone.0159086.s003.pdf]

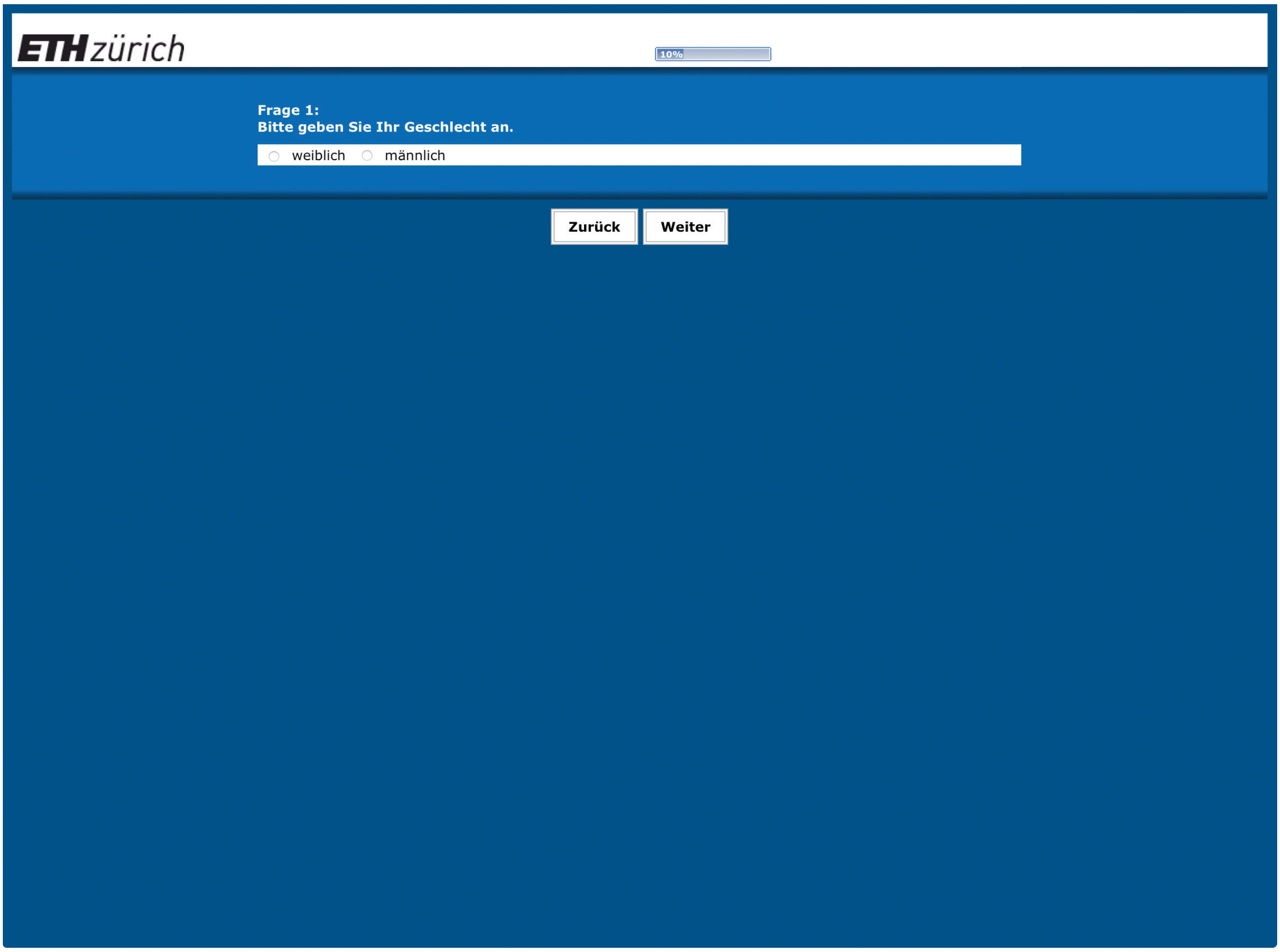

Question 1: “Please tell us your gender – female/male”

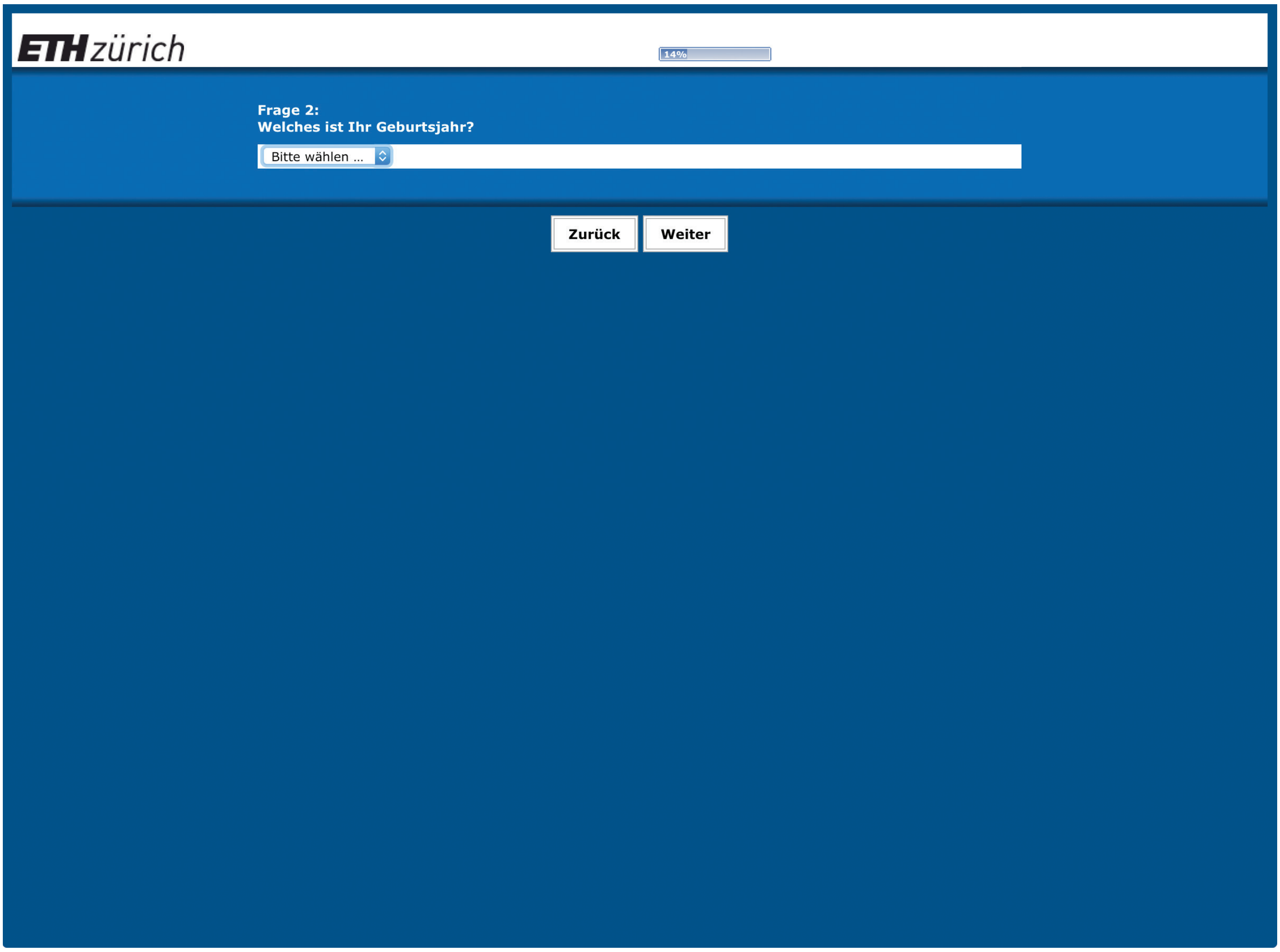

Question 2: “What is your year of birth?”

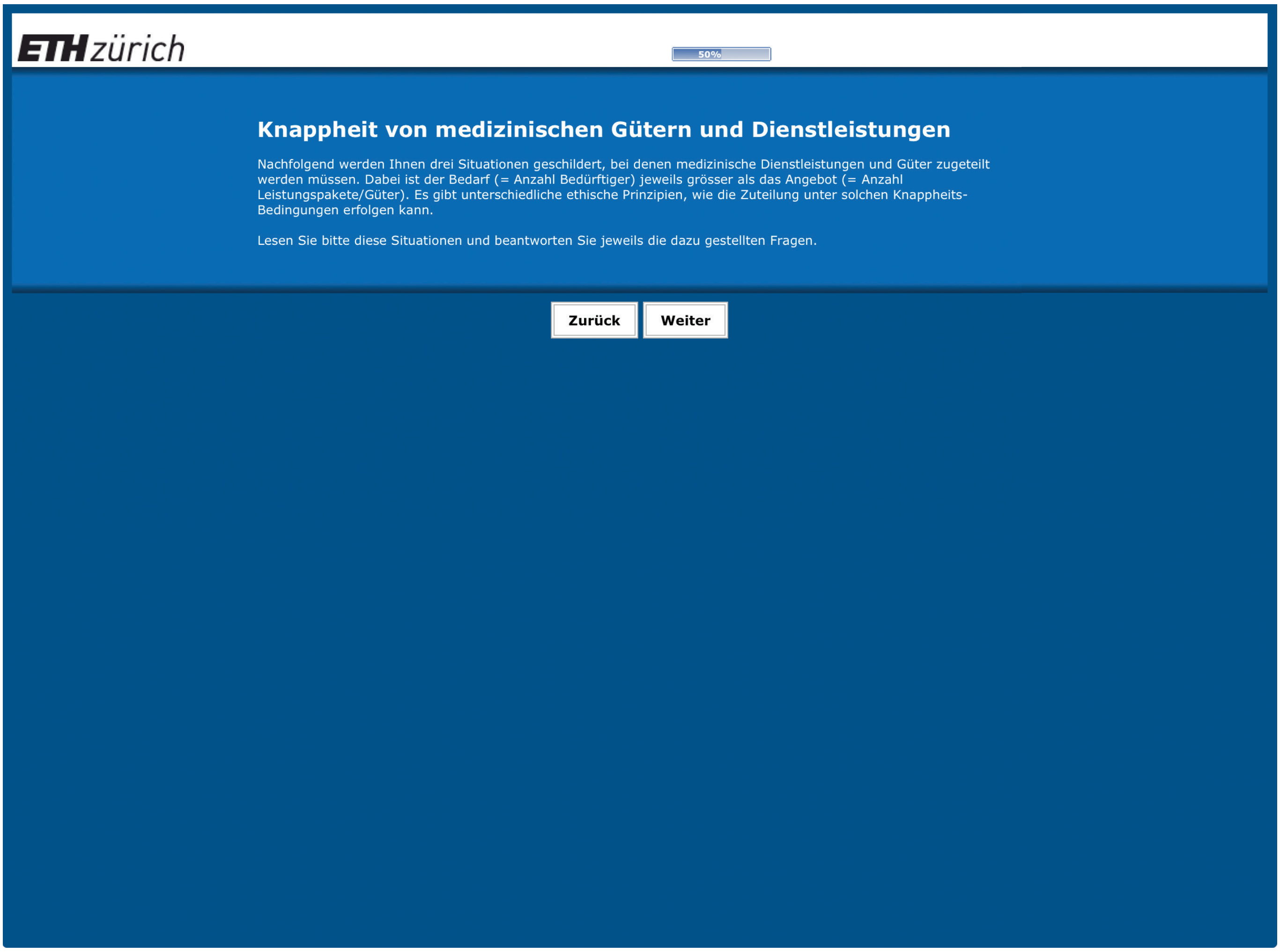

Scarcity of medical goods and services

On the following pages, three situations will be presented to you, in which scarce medical goods and services need to be allocated. The situations are characterized by the fact that demand (= number of those in need) is higher than supply (= number of service packages / goods). There are different ethical principles that may guide the allocation of resources under such conditions of scarcity.

Please read the descriptions of the situations and answer the respective questions.

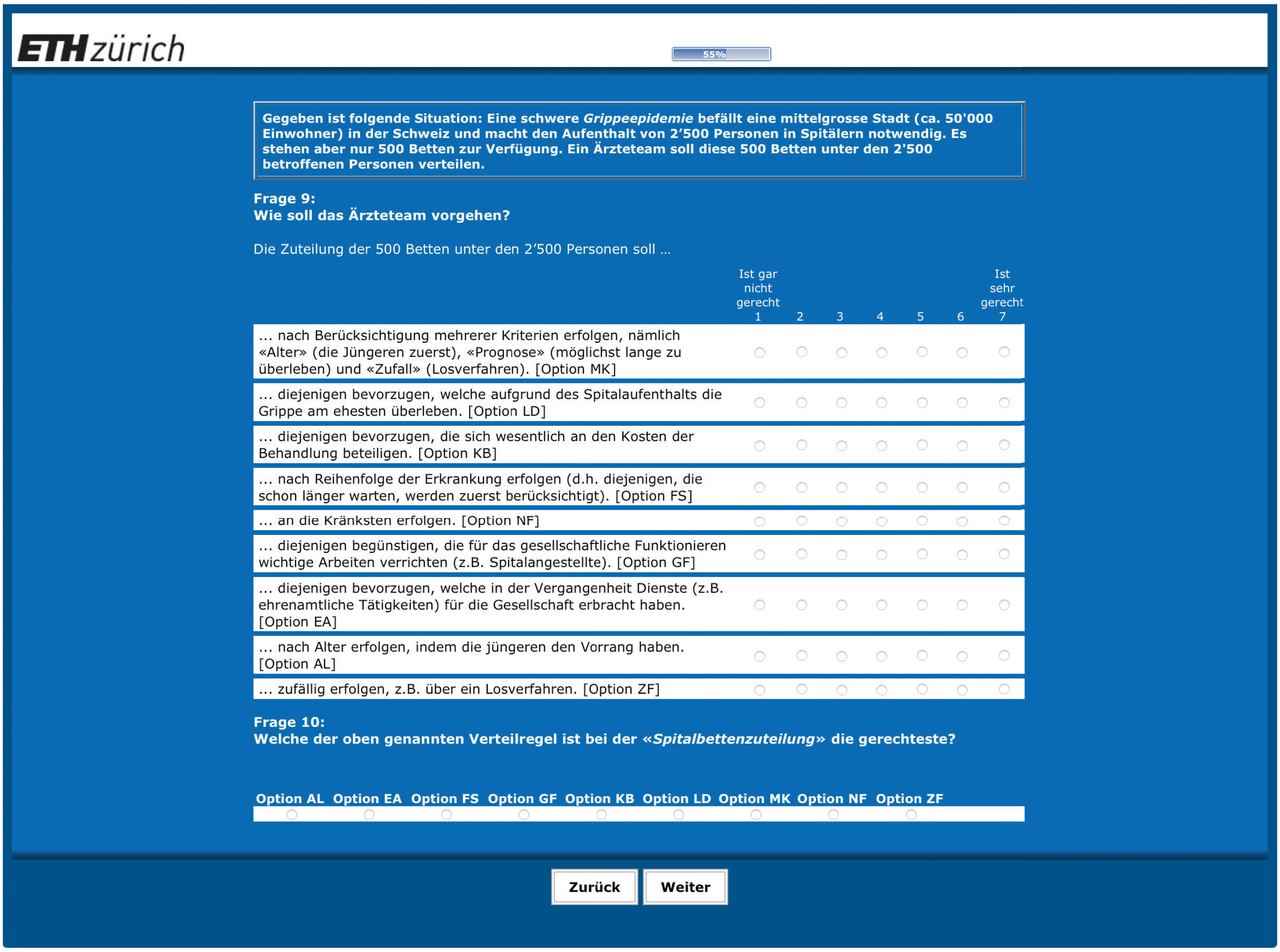

ETH zürich

55%

Gegeben ist folgende Situation: Eine schwere *Grippeepidemie* befällt eine mittelgrosse Stadt (ca. 50'000 Einwohner) in der Schweiz und macht den Aufenthalt von 2'500 Personen in Spitälern notwendig. Es stehen aber nur 500 Betten zur Verfügung. Ein Ärzteteam soll diese 500 Betten unter den 2'500 betroffenen Personen verteilen.

Frage 9:
Wie soll das Ärzteteam vorgehen?

Die Zuteilung der 500 Betten unter den 2'500 Personen soll ...

	Ist gar nicht gerecht 1	2	3	4	5	6	Ist sehr gerecht 7
... nach Berücksichtigung mehrerer Kriterien erfolgen, nämlich «Alter» (die Jüngeren zuerst), «Prognose» (möglichst lange zu überleben) und «Zufall» (Losverfahren). [Option MK]	○	○	○	○	○	○	○
... diejenigen bevorzugen, welche aufgrund des Spitalaufenthalts die Grippe am ehesten überleben. [Option LD]	○	○	○	○	○	○	○
... diejenigen bevorzugen, die sich wesentlich an den Kosten der Behandlung beteiligen. [Option KB]	○	○	○	○	○	○	○
... nach Reihenfolge der Erkrankung erfolgen (d.h. diejenigen, die schon länger warten, werden zuerst berücksichtigt). [Option FS]	○	○	○	○	○	○	○
... an die Kränksten erfolgen. [Option NF]	○	○	○	○	○	○	○
... diejenigen begünstigen, die für das gesellschaftliche Funktionieren wichtige Arbeiten verrichten (z.B. Spitalangestellte). [Option GF]	○	○	○	○	○	○	○
... diejenigen bevorzugen, welche in der Vergangenheit Dienste (z.B. ehrenamtliche Tätigkeiten) für die Gesellschaft erbracht haben. [Option EA]	○	○	○	○	○	○	○
... nach Alter erfolgen, indem die jüngeren den Vorrang haben. [Option AL]	○	○	○	○	○	○	○
... zufällig erfolgen, z.B. über ein Losverfahren. [Option ZF]	○	○	○	○	○	○	○

Frage 10:
Welche der oben genannten Verteilregel ist bei der «*Spitalbettenzuteilung*» die gerechteste?

Option AL	Option EA	Option FS	Option GF	Option KB	Option LD	Option MK	Option NF	Option ZF
○	○	○	○	○	○	○	○	○

Zurück Weiter

Questions 9 & 10: Situation *hospital beds*

Translation of description and questions is provided in Table 2. Acronyms used in the online survey correspond to those in Table 2 of the bread text: NF=SICK; FS=ORDR; LD=SURV; GF=IMPF; MK=COMB; AL=YONG; ZF=RAND; EA=SERV; KB=MONY. 7-point Likert scales were used ranging from 1=totally unjust to 7=totally just.

Question 10: Which of the before mentioned allocation principles is the fairest?

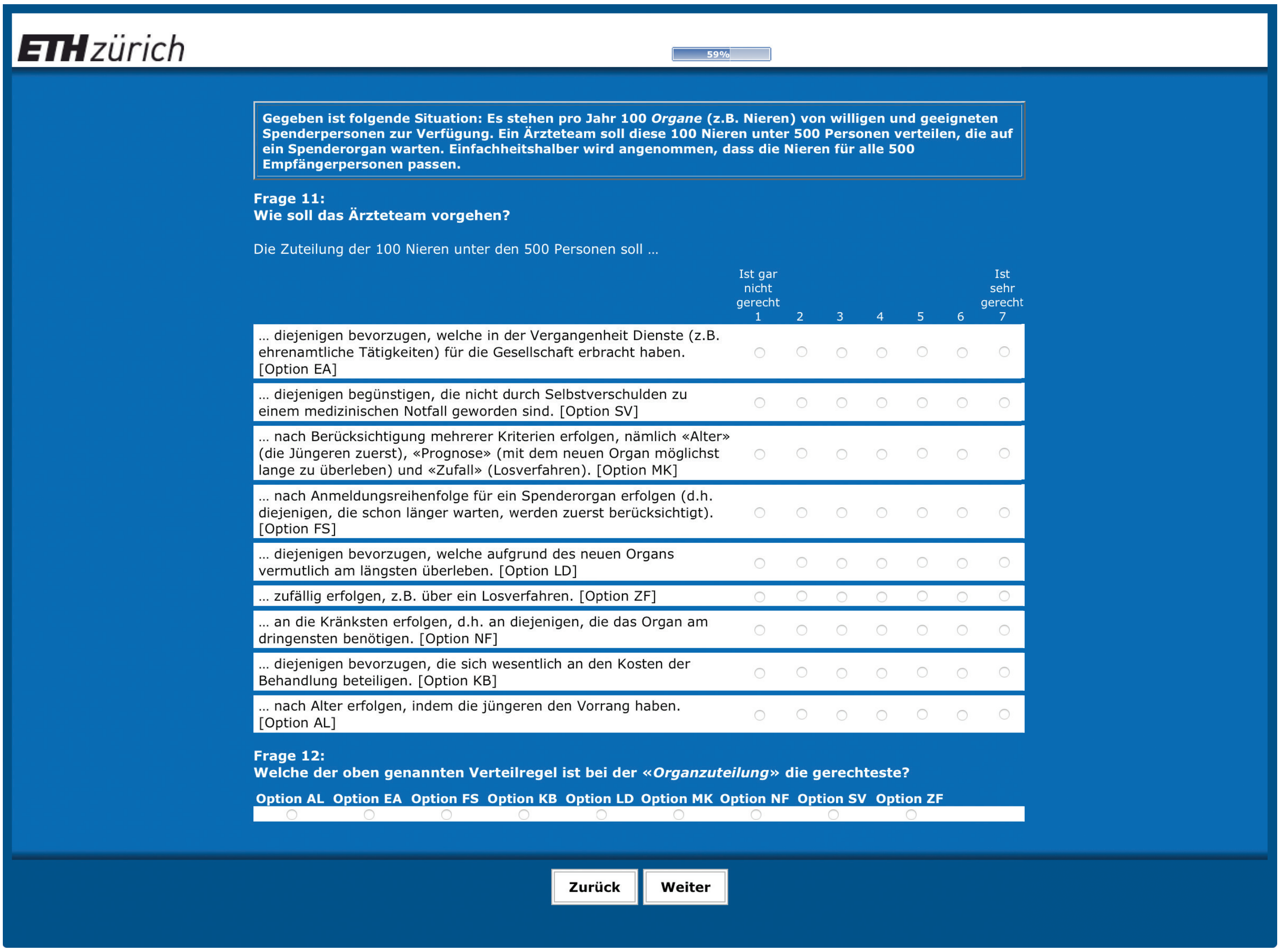

ETH zürich

59%

Gegeben ist folgende Situation: Es stehen pro Jahr 100 *Organe* (z.B. Nieren) von willigen und geeigneten Spenderpersonen zur Verfügung. Ein Ärzteteam soll diese 100 Nieren unter 500 Personen verteilen, die auf ein Spenderorgan warten. Einfachheitshalber wird angenommen, dass die Nieren für alle 500 Empfängerpersonen passen.

Frage 11:
Wie soll das Ärzteteam vorgehen?

Die Zuteilung der 100 Nieren unter den 500 Personen soll …

	Ist gar nicht gerecht 1	2	3	4	5	6	Ist sehr gerecht 7
… diejenigen bevorzugen, welche in der Vergangenheit Dienste (z.B. ehrenamtliche Tätigkeiten) für die Gesellschaft erbracht haben. [Option EA]							
… diejenigen begünstigen, die nicht durch Selbstverschulden zu einem medizinischen Notfall geworden sind. [Option SV]							
… nach Berücksichtigung mehrerer Kriterien erfolgen, nämlich «Alter» (die Jüngeren zuerst), «Prognose» (mit dem neuen Organ möglichst lange zu überleben) und «Zufall» (Losverfahren). [Option MK]							
… nach Anmeldungsreihenfolge für ein Spenderorgan erfolgen (d.h. diejenigen, die schon länger warten, werden zuerst berücksichtigt). [Option FS]							
… diejenigen bevorzugen, welche aufgrund des neuen Organs vermutlich am längsten überleben. [Option LD]							
… zufällig erfolgen, z.B. über ein Losverfahren. [Option ZF]							
… an die Kränksten erfolgen, d.h. an diejenigen, die das Organ am dringensten benötigen. [Option NF]							
… diejenigen bevorzugen, die sich wesentlich an den Kosten der Behandlung beteiligen. [Option KB]							
… nach Alter erfolgen, indem die jüngeren den Vorrang haben. [Option AL]							

Frage 12:
Welche der oben genannten Verteilregel ist bei der «*Organzuteilung*» die gerechteste?

Option AL	Option EA	Option FS	Option KB	Option LD	Option MK	Option NF	Option SV	Option ZF

Zurück Weiter

Questions 11 & 12: Situation *donor organs*

Translation of description and questions is provided in Table 2. Acronyms used in the online survey correspond to those in Table 2 of the bread text: NF=SICK; FS=ORDR; LD=SURV; SV=BHAV; MK=COMB; AL=YONG; ZF=RAND; EA=SERV; KB=MONY. 7-point Likert scales were used ranging from 1=totally unjust to 7=totally just.

Question 12: Which of the before mentioned allocation principles is the fairest?

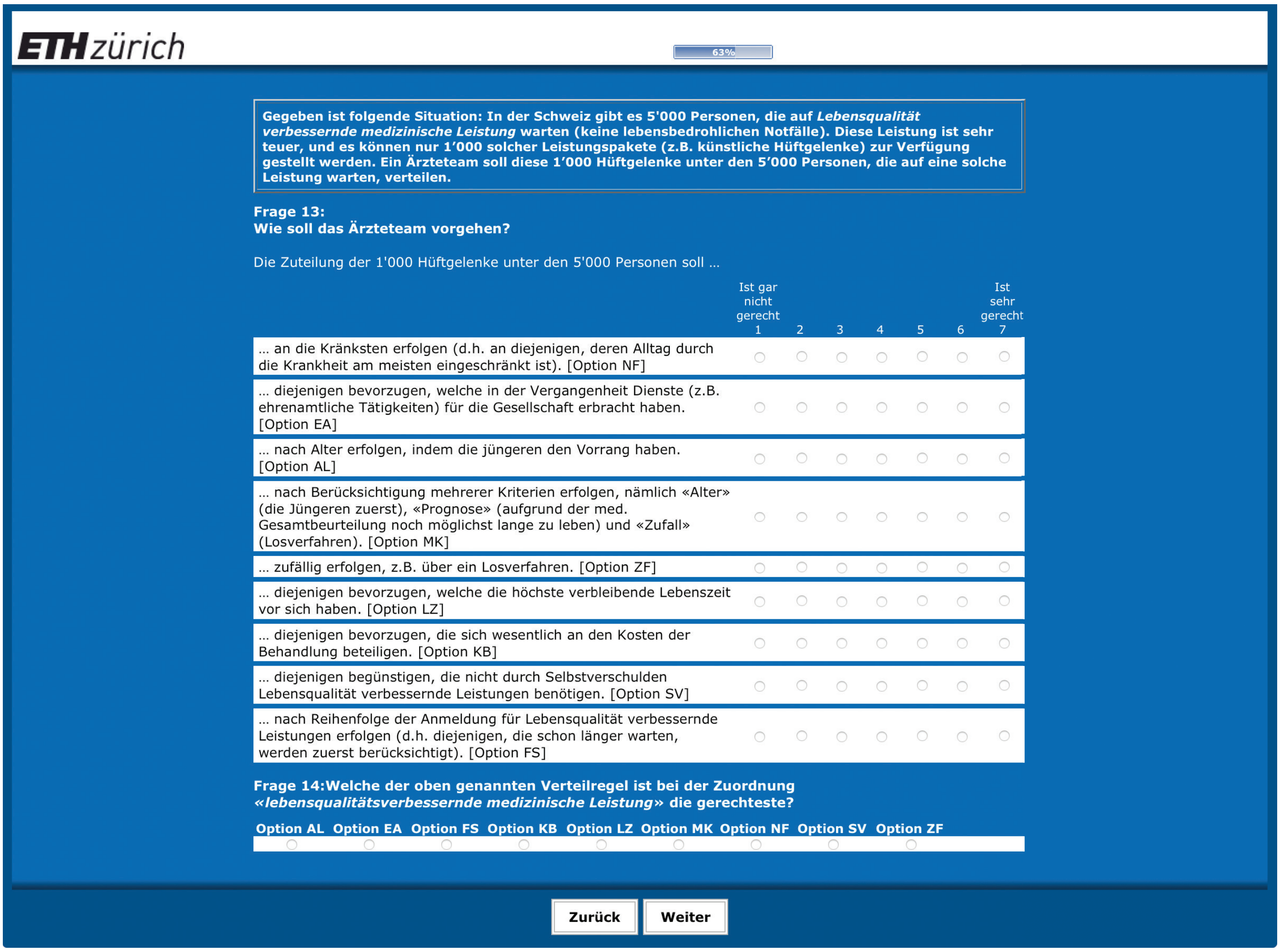

ETHzürich

63%

Gegeben ist folgende Situation: In der Schweiz gibt es 5'000 Personen, die auf *Lebensqualität verbessernde medizinische Leistung* warten (keine lebensbedrohlichen Notfälle). Diese Leistung ist sehr teuer, und es können nur 1'000 solcher Leistungspakete (z.B. künstliche Hüftgelenke) zur Verfügung gestellt werden. Ein Ärzteteam soll diese 1'000 Hüftgelenke unter den 5'000 Personen, die auf eine solche Leistung warten, verteilen.

Frage 13:
Wie soll das Ärzteteam vorgehen?

Die Zuteilung der 1'000 Hüftgelenke unter den 5'000 Personen soll …

	Ist gar nicht gerecht 1	2	3	4	5	6	Ist sehr gerecht 7
… an die Kränksten erfolgen (d.h. an diejenigen, deren Alltag durch die Krankheit am meisten eingeschränkt ist). [Option NF]							
… diejenigen bevorzugen, welche in der Vergangenheit Dienste (z.B. ehrenamtliche Tätigkeiten) für die Gesellschaft erbracht haben. [Option EA]							
… nach Alter erfolgen, indem die jüngeren den Vorrang haben. [Option AL]							
… nach Berücksichtigung mehrerer Kriterien erfolgen, nämlich «Alter» (die Jüngeren zuerst), «Prognose» (aufgrund der med. Gesamtbeurteilung noch möglichst lange zu leben) und «Zufall» (Losverfahren). [Option MK]							
… zufällig erfolgen, z.B. über ein Losverfahren. [Option ZF]							
… diejenigen bevorzugen, welche die höchste verbleibende Lebenszeit vor sich haben. [Option LZ]							
… diejenigen bevorzugen, die sich wesentlich an den Kosten der Behandlung beteiligen. [Option KB]							
… diejenigen begünstigen, die nicht durch Selbstverschulden Lebensqualität verbessernde Leistungen benötigen. [Option SV]							
… nach Reihenfolge der Anmeldung für Lebensqualität verbessernde Leistungen erfolgen (d.h. diejenigen, die schon länger warten, werden zuerst berücksichtigt). [Option FS]							

Frage 14:Welche der oben genannten Verteilregel ist bei der Zuordnung *«lebensqualitätsverbessernde medizinische Leistung»* die gerechteste?

Option AL	Option EA	Option FS	Option KB	Option LZ	Option MK	Option NF	Option SV	Option ZF

Zurück Weiter

Questions 13 & 14: Situation *joint replacements*

Translation of description and questions is provided in Table 2. Acronyms used in the online survey correspond to those in Table 2 of the bread text: NF=SICK; FS=ORDR; LZ=SURV; SV=BHAV; MK=COMB; AL=YONG; ZF=RAND; EA=SERV; KB=MONY. 7-point Likert scales were used, ranging from 1=totally unjust to 7=totally just.

Question 14: Which of the before mentioned allocation principles is the fairest?

ETH zürich

79%

Lebenssituation

Frage 20:
Zufriedenheit
Wie zufrieden sind Sie insgesamt …

	äusserst unzufrieden 1	2	3	4	5	6	äusserst zufrieden 7
… gegenwärtig mit Ihrem Leben?	○	○	○	○	○	○	○
… mit der aktuellen wirtschaftlichen Lage in der Schweiz?	○	○	○	○	○	○	○

Frage 21:
Gesundheit

	sehr schlecht 1	2	3	4	5	6	sehr gut 7
Wie stufen Sie Ihren allgemeinen Gesundheitszustand ein?	○	○	○	○	○	○	○

Frage 22:
Einschränkung der täglichen Aktivitäten

	Ja, sehr stark 1	Ja, bis zu einem gewissen Grad 2	Nein 3
Werden Sie bei Ihren täglichen Aktivitäten in irgendeiner Form durch eine langwierige körperliche oder seelische Krankheit oder eine Behinderung eingeschränkt?	○	○	○

Zurück **Weiter**

Question 21: Health

How would you rate your current health state? Very bad (1) … Very good (7)

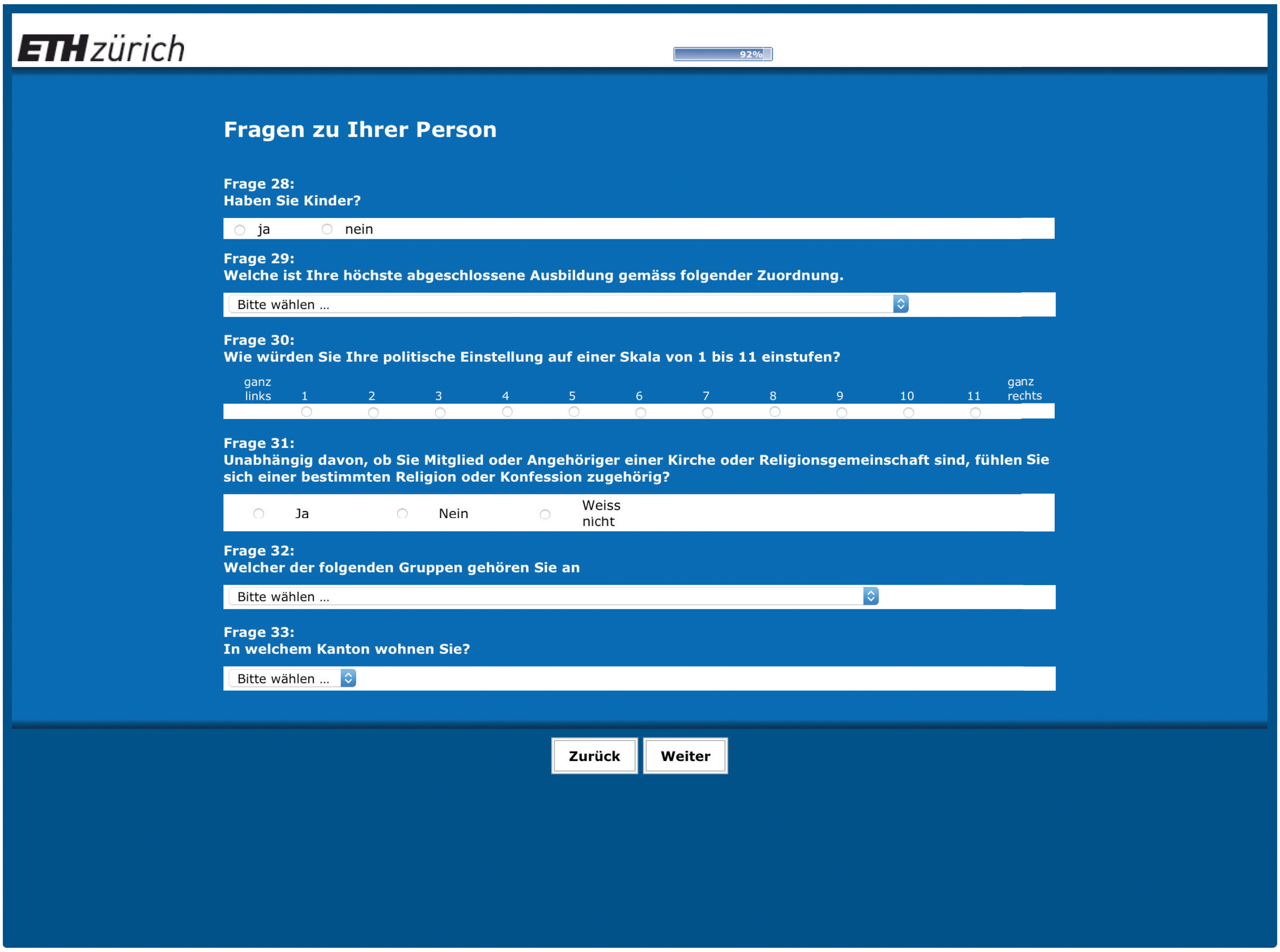

Question 30: Political orientation

How would you rate your political orientation on a scale ranging from 1 (totally left oriented) to 11 (totally right oriented).

Question 31: Religion

Independent of whether you are member of a church or religious community do you feel connected to a specific religion or confession? Yes/no/don’t know.

Question 32: Group

Which of the following groups do you belong to:
- medical doctor
- medical student
- other health professionals
- none of the before mentioned groups
