# Supplementary material for: How to Fairly Allocate Scarce Medical Resources: Ethical Argumentation under Scrutiny by Health Professionals and Lay People
Source: PLoS One. 2016 Jul 27;11(7):e0159086. doi: 10.1371/journal.pone.0159086 (PMC4963105; doi:10.1371/journal.pone.0159086)
Supplement: S1 Table — a Percentage; b 11-point scales ranging from 1 = most left to 11 = most right. (DOC) [file pone.0159086.s004.doc]

S1 Table. Socio-demographic profile of respondent groups.

| **Respondents** | **Number** | **Age** | **Gender**^a^ | **Political orientation**^b^ | **Religiosity**^a^ |
| --- | --- | --- | --- | --- | --- |
| **Groups** |  | **Mean (SD)** | **Female / male** | **Mean (SD)** | **Religious / non-religious / undecided** |
| LP | 822 | 44.7 (10.7) | 47.4 / 52.6 | 6.0 (2.2) | 47.8 / 45.1 / 7.1 |
| GP | 212 | 55.3 (9.4) | 26.9 / 73.1 | 5.3 (2.2) | 67.0 / 27.8 / 5.2 |
| MS | 171 | 23.4 (5.0) | 67.3 / 32.7 | 4.7 (2.1) | 37.4 / 52.6 / 9.9 |
| HP | 62 | 44.4 (11.2) | 83.9 / 16.1 | 5.6 (1.9) | 51.6 / 38.7 / 9.7 |

^a^ Percentage

^b^ 11-point scales ranging from 1= most left to 11= most right.
