# Supplementary material for: How to Fairly Allocate Scarce Medical Resources: Ethical Argumentation under Scrutiny by Health Professionals and Lay People
Source: PLoS One. 2016 Jul 27;11(7):e0159086. doi: 10.1371/journal.pone.0159086 (PMC4963105; doi:10.1371/journal.pone.0159086)
Supplement: S2 Table — 7-point Likert scales ranging from 1 = totally unjust to 7 = totally just. (DOC) [file pone.0159086.s005.doc]

**S2 Table. Mean (M), standard deviation (SD), F-statistic, and p-values of fairness ratings of nine allocation principles by medical students, general practitioners, other health professionals, and lay people for three situations of scarce medical resource allocations.**

| **Allocation**  **principle** | **Medical background** | | | | | | | | **Test statistics (Anova)** | |
| --- | --- | --- | --- | --- | --- | --- | --- | --- | --- | --- |
|  | **Medical students (N=171)** | | **General practitioners (N=212)** | | **Other health professionals (N=62)** | | **Lay people (N=822)** | | **F-statistic** | **p-value** |
|  | **M** | **SD** | **M** | **SD** | **M** | **SD** | **M** | **SD** |  |  |
| **Situation A:** Inelastic / frequent events (donor organs) | | | | | | | | | | |
| SICK | 5.56 | 1.251 | 5.15 | 1.653 | 5.40 | 1.336 | 5.82 | 1.385 | 14.032 | 0.000 |
| ORDR | 4.73 | 1.434 | 4.21 | 1.749 | 4.85 | 1.389 | 5.14 | 1.571 | 20.365 | 0.000 |
| SURV | 5.45 | 1.368 | 5.33 | 1.416 | 5.16 | 1.462 | 4.40 | 1.695 | 34.809 | 0.000 |
| BHAV | 4.40 | 1.767 | 3.84 | 1.776 | 4.63 | 1.739 | 4.18 | 1.874 | 4.393 | 0.004 |
| COMB | 5.20 | 1.529 | 5.18 | 1.616 | 4.74 | 1.774 | 3.96 | 1.872 | 42.090 | 0.000 |
| YONG | 4.19 | 1.411 | 4.48 | 1.592 | 4.29 | 1.740 | 3.65 | 1.710 | 18.097 | 0.000 |
| RAND | 2.97 | 1.764 | 2.73 | 1.779 | 2.48 | 1.607 | 2.43 | 1.761 | 5.378 | 0.001 |
| SERV | 2.51 | 1.399 | 2.16 | 1.336 | 2.23 | 1.487 | 2.37 | 1.531 | 2.014 | 0.110 |
| MONY | 2.04 | 1.317 | 2.08 | 1.385 | 2.02 | 1.349 | 2.42 | 1.551 | 5.821 | 0.001 |
| **Situation B:** Inelastic / rare events (hospital beds) | | | | | | | | | | |
| SICK | 5.80 | 1.188 | 5.31 | 1.622 | 5.66 | 1.366 | 5.69 | 1.481 | 4.521 | 0.004 |
| ORDR | 3.98 | 1.553 | 3.23 | 1.741 | 4.89 | 1.415 | 4.83 | 1.667 | 58.706 | 0.000 |
| SURV | 5.25 | 1.560 | 5.25 | 1.593 | 4.45 | 1.939 | 4.10 | 1.781 | 38.008 | 0.000 |
| IMPF | 3.63 | 1.728 | 3.95 | 1.830 | 3.76 | 1.939 | 3.41 | 1.842 | 5.299 | 0.001 |
| COMB | 4.61 | 1.632 | 4.49 | 1.892 | 4.45 | 1.997 | 3.69 | 1.832 | 20.433 | 0.000 |
| YONG | 3.17 | 1.479 | 3.50 | 1.735 | 3.60 | 1.541 | 3.14 | 1.654 | 3.937 | 0.008 |
| RAND | 2.91 | 1.752 | 2.21 | 1.581 | 2.61 | 1.832 | 2.43 | 1.790 | 5.411 | 0.001 |
| SERV | 2.40 | 1.420 | 2.08 | 1.293 | 2.60 | 1.562 | 2.39 | 1.547 | 3.221 | 0.022 |
| MONY | 2.13 | 1.286 | 1.87 | 1.277 | 2.19 | 1.365 | 2.46 | 1.563 | 10.166 | 0.000 |
| **Situation C** Elastic / frequent events (joint replacement) | | | | | | | | | | |
| SICK | 5.87 | 1.000 | 5.60 | 1.229 | 5.63 | 1.177 | 5.63 | 1.462 | 1.540 | 0.202 |
| ORDR | 4.71 | 1.362 | 4.14 | 1.725 | 4.66 | 1.330 | 4.79 | 1.670 | 9.031 | 0.000 |
| SURV | 4.70 | 1.510 | 4.59 | 1.556 | 4.31 | 1.636 | 3.86 | 1.777 | 18.926 | 0.000 |
| BHAV | 4.18 | 1.717 | 3.75 | 1.755 | 4.42 | 1.751 | 4.15 | 1.812 | 3.730 | 0.011 |
| COMB | 5.12 | 1.436 | 4.83 | 1.641 | 4.68 | 1.845 | 3.76 | 1.834 | 43.297 | 0.000 |
| YONG | 4.20 | 1.470 | 4.13 | 1.611 | 3.82 | 1.760 | 3.46 | 1.707 | 15.645 | 0.000 |
| RAND | 3.10 | 1.840 | 2.57 | 1.787 | 2.55 | 1.844 | 2.49 | 1.788 | 5.439 | 0.001 |
| SERV | 2.63 | 1.393 | 2.41 | 1.459 | 2.60 | 1.509 | 2.45 | 1.537 | 0.981 | 0.401 |
| MONY | 2.67 | 1.571 | 2.56 | 1.678 | 2.50 | 1.479 | 2.70 | 1.668 | 0.590 | 0.622 |

7-point Likert scales ranging from 1=totally unjust to 7=totally just.
