# Supplementary material for: How to Fairly Allocate Scarce Medical Resources: Ethical Argumentation under Scrutiny by Health Professionals and Lay People
Source: PLoS One. 2016 Jul 27;11(7):e0159086. doi: 10.1371/journal.pone.0159086 (PMC4963105; doi:10.1371/journal.pone.0159086)
Supplement: S1 Text — (DOCX) [file pone.0159086.s006.docx]

**S1 Text. Codebook of dataset used for the current study.**

Demographic variables

Gender 1 female

2 male

Age [in years; only year of birth, not exact date was known]

HealthState 1 very bad

2

3

4

5

6

7 very good

PoliticalOrientation 1 left

2

3

4

5

6

7

8

9

10

11 right

Religion 1 yes

2 no

3 don't know

MedicalBackground 1 student

2 practitioner

3 other health professional

4 lay person

RespondentPool 1 market research panel

2 general practitioners

3 student mailing list

Fairness ratings and rankings

S1_ donor organs

S2_ hospital beds

S3_ joint replacements

_SICK, _ORDR, _SURV, _BHAV, _COMB, _YONG, _RAND, _SERV, _MONY, _IMPF cf. Table 2, main text of the paper

Fairness ratings 1 totally unfair

2

3

4

5

6

7 totally fair

S1_Fairest 1 YONG

2 SERV

3 ORDR

4 MONY

5 SURV

6 COMB

7 SICK

8 BHAV

9 RAND

S2_Fairest 1 YONG

2 SERV

3 ORDR

4 IMPF

5 MONY

6 SURV

7 COMB

8 SICK

9 RAND

S3_Fairest 1 YONG

2 SERV

3 ORDR

4 MONY

5 SURV

6 COMB

7 SICK

8 BHAV

9 RAND
